# Accurate 3D recording: Integrating ground-based LiDAR data and 3D segmentation network to extract 3D traits and analyze genetics in wheat populations

**DOI:** 10.1016/j.plaphe.2026.100229

**Published:** 2026-06-01

**Authors:** Honghao Zhou, Bingxi Qin, Chunmei Guo, Jingrong Zang, An Wang, Yin Li, Zijun Pei, Qi Sun, Qiang Zheng, Qing Li, Dong Jiang, Jiawei Chen

**Affiliations:** Academy for Advanced Interdisciplinary Studies, Collaborative Innovation Centre for Modern Crop Production, Co-sponsored by Province and Ministry, College of Agriculture, State Key Laboratory of Crop Genetics & Germplasm Enhancement and Utilization, Nanjing Agricultural University, Nanjing, 210095, China

**Keywords:** Wheat growing period, 3D point cloud, 3D segmentation network, 3D phenotypic parameter, Genome-wide association study

## Abstract

This study suggests a novel extraction pipeline based on terrestrial laser scanning across multiple growth stages to address the current deficiency of three-dimensional (3D) phenotypic traits for wheat populations derived from 3D point clouds. This study presents 3D Wheat Point-seg Net (3D WP-seg Net), a novel 3D point cloud segmentation network that incorporates an SA-CrossAttention module to address the difficulties presented by complex structures, background noise, non-uniform point distributions, and scale variations in plot-level wheat point cloud data. Plot height, canopy area, and volume are examples of common phenotypic parameters that are successfully extracted using this technique. Additionally, two new phenotypic parameters: plot extension distance and lodging angle are suggested by fusing the centroid and slice-skeletonization algorithms. A software platform called 3D Trait Analysis was created to facilitate multi-sensor 3D data processing and trait extraction. A genome-wide association study (GWAS) was then conducted using the extracted population-level traits to find potential genes linked to these new phenotypes. While the segmentation accuracies of 3D WP-seg Net achieved 93.1%, 88.3%, and 92.5% under various sensor systems, the results showed a strong correlation between the predicted and measured plot heights (R^2^ = 0.954). Furthermore, four candidate genes linked to extension distance were found on chromosomes 1A, 2A, and 4A, and five putative genes controlling plot lodging angle were found on chromosomes 2D, 3A, and 7A. The multi-stage 3D phenotyping and analysis framework for wheat populations established by this study improves the accuracy of point cloud segmentation and trait quantification while offering a new and efficient method for the genetic analysis of important population-level traits.

## 1 Introduction

One of the most extensively grown food crops in the world, wheat contributes significantly to global food security [1]. Wheat's phenotypic characteristics show how the plant has adapted to the different environmental factors and management techniques that affect its growth process [2]. In addition to providing crucial evidence for genome-phenotype association analysis, phenotypic data also aid in revealing dynamic changes in crop growth [3]. Thus, a thorough examination of wheat's phenotypic traits can reveal its growth trends and offer vital information for crop management and yield forecasting [4]. Manual measurements and visual inspections are examples of traditional phenotyping techniques that are frequently laborious and prone to human error [5]. Despite the fact that 2D data analysis techniques are frequently used in phenotypic research, they are unable to accurately depict plant canopy structure, volume, and spatial relationships due to their lack of depth information. Therefore, when processing complex plant structures, 2D data has significant limitations [6,7]. The advancement of 3D imaging technology has made it possible to obtain rich spatial information and precisely capture the intricate structure of crops using 3D point cloud data. In contrast to 2D data, it accurately depicts the distribution and interactions of plants in space in addition to revealing the crop's appearance [8]. This technique can effectively and precisely extract important wheat phenotypic traits, offering solid support for evaluations of crop health and growth conditions.

3D data of crop populations can be obtained outdoors using a variety of techniques, each with pros and cons depending on the application. By taking images from various perspectives, drone-based multi-view imaging creates three-dimensional (3D) models of crops [[9], [10], [11]]. This approach has limitations in terms of obtaining detailed trait parameters, despite its high data collection efficiency and ability to effectively cover large-scale field areas. Capturing fine-grained traits is challenging because of drone camera resolution and shooting angle limitations, as well as the inability to provide enough detail in regions with taller crops or dense plant populations. The backpack radar system is appropriate for challenging field terrains because it employs portable devices to collect point clouds in a flexible manner [12,13]. However, it is challenging to precisely capture details in areas with dense canopies or overlapping plants because of its lower resolution and scanning density, particularly when crop population analysis calls for high precision. On the other hand, LiDAR (Light Detection and Ranging) systems offer a number of benefits when it comes to gathering 3D point cloud data about crop populations. LiDAR can provide high-precision phenotypic data by penetrating plant cover with laser beams and receiving reflected signals. This enables precision agriculture applications and high-precision phenotypic analysis [[14], [15], [16]].

Crop population phenotypic analysis has advanced significantly with the use of 3D point cloud segmentation technology, particularly in high-precision segmentation and complex environment handling. Conventional 3D point cloud segmentation algorithms work well in simplified settings. Examples include the DBSCAN algorithm for plant recognition in soybean fields [17], the K-means clustering algorithm for maize canopy segmentation [18], and plane-fitting-based segmentation to eliminate ground points and preserve crop points [19]. However, these techniques frequently fail to provide accurate segmentation and are prone to either over- or under-segmentation in complex field environments, particularly in high-density crop populations or overlapping plants. Furthermore, the local geometric structure of point clouds is not well understood by conventional methods, which makes it challenging to handle interference from environmental noise and dynamic canopy changes. For processing intricate 3D point clouds of crop populations, deep learning-based point cloud segmentation network architectures have progressively taken the lead in recent years [20]. By automatically extracting sophisticated features from point clouds using deep learning, point cloud segmentation networks greatly increase segmentation accuracy and successfully handle occlusion and noise problems in complex environments [21]. For instance [22], segmented the aboveground portion of wheat populations using PointNet++, and [23] detected and segmented point clouds of vegetable crop populations using DGCNN. By learning the spatial relationships and geometric features of point clouds, these deep learning-based networks overcome the drawbacks of conventional algorithms in high-density or occlusion scenarios, greatly enhancing the efficiency of crop population segmentation.

Several important 3D structural traits can be extracted for additional phenotypic analysis once precise crop population point clouds have been obtained using 3D point cloud segmentation networks. Canopy height is the most fundamental phenotypic indicator among these 3D structural features. The P_90_ or P_95_ height quantiles that are taken from 3D point clouds have been used extensively for population growth tracking and growth stage assessment. They are correlated with manual measurements and above-ground biomass height [[24], [25], [26], [27]]. Point clouds can be projected onto the ground plane to create a canopy projection area or coverage. This indicator is used to assess population structure uniformity in a variety of crops and represents the crop population's capacity to intercept light energy [[28], [29], [30]]. To build non-destructive biomass or yield prediction models, canopy volume (or the volume occupied by the population) is a common 3D structural parameter that has been demonstrated to be highly correlated with canopy photosynthetic potential and yield performance in crops like rice and maize [31,32]. Furthermore, point cloud canopy thickness and leaf density distribution can be used to indirectly estimate the leaf area index (LAI), a key measure of population photosynthetic capacity. Field crops now have an inversion correlation for LAI of R^2^ ~ 0.8, which provides crucial input parameters for precision farming [[33], [34], [35]]. In addition to aiding in the fine-grained characterization of the population posture and spatial structural evolution of wheat populations, certain traits like lodging angle, stretch length, and canopy density also improve the capacity to depict responses to stress and cultivation practices. This offers more precise and measurable phenotypic support for field precision management, density and nitrogen fertilizer optimization, and breeding for wheat lodging resistance.

With the following four primary goals in mind, this study attempts to develop an automated trait extraction framework for wheat populations based on ground-based LiDAR-derived 3D point clouds to address these problems.

1. To accomplish accurate segmentation of background point clouds and wheat plots, suggest the 3D WP-seg Net point cloud segmentation network.
2. Using centroid calculations and slice skeletonization techniques, this study further constructs two new population structural traits: plot extension distance and lodging angle based on the conventional phenotypic indicators of plot height, canopy area, and volume.
3. Create software for 3D trait analysis: Automate every step of the process, from reading and processing multi-source crop population point clouds to producing results, by integrating 3D visualization, automatic segmentation, and phenotypic extraction functions.
4. GWAS analysis and potential gene mining: Determine possible genes linked to the suggested two population traits by performing a genome-wide association analysis of particular varieties at different growth stages.

## 2 Materials and methods

### 2.1 Natural wheat population field experiments

**Fig. 1:**
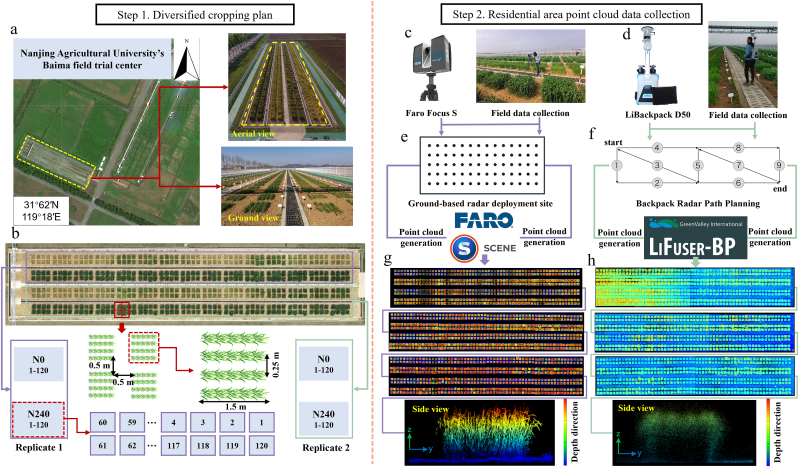
The natural wheat population's field layout and data collection. (a) A summary of the field of experimentation. (b) The wheat population plot layout. (c) Setup for terrestrial laser scanning. (d) Setting up a backpack LiDAR scanner. (e) A scanning layout that is fixed in place. (f) Triangular-path scanning route on a mobile device. (g) Terrestrial laser scanner point cloud. (h) Point cloud of the Backpack LiDAR system.

Using 120 natural wheat varieties gathered from the Huang–Huai–Hai Plain and the middle and lower reaches of the Yangtze River as experimental materials, this study was carried out between 2022 and 2023. The Baimai Teaching and Research Base in Lishui District, Nanjing, Jiangsu Province, China (31°62′N, 119°18′E) was the site of field tests (Fig. 1a). A high-nitrogen (N240) and zero-nitrogen (N0) nitrogen fertilizer treatments were created. A total of 480 independent experimental plots were produced, with two replicates for each nitrogen level to guarantee the validity and reliability of the experimental findings (Fig. 1b). For even sowing, each plot was 1.5 m by 1 m and had rows spaced 25 cm apart. There were four rows per plot, with about 100 wheat plants planted in each row. Following seeding, each plot received consistent irrigation at a rate of about 20 L per square meter. To avoid an overabundance of water, irrigation volumes were controlled based on local rainfall conditions, typically remaining between 5% and 15% of the natural precipitation. By maintaining consistent soil moisture and uniform field management, this ensured an accurate assessment of nitrogen effects.

### 2.2 Acquiring 3D point cloud data of wheat populations at various growth stages

A LiBackpack D50 mobile laser scanning system (GreenValley International Ltd., Beijing, China) and a FARO Focus S series terrestrial laser scanner (FARO Technologies Inc., FL, USA) were used to obtain 3D point cloud data of the wheat canopy. The terrestrial laser scanner (Fig. 1c) is 230 × 183 × 103 mm in size and weighs about 4.2 kg. With a scanning field of view of 360° horizontally and 300° vertically, it functions at a wavelength of 1550 nm. With a typical noise level of 0.30 mm at a distance of 10 m, the measurement range begins at 0.6 m. With the help of two Velodyne VLP-16E laser sensors (Velodyne Lidar Inc., San Jose, CA, USA), an IMU (Inertial Measurement Unit), and a GNSS positioning module, the LiBackpack D50 (Fig. 1d) can perform simultaneous localization and mapping (SLAM) while in motion. The system is appropriate for field mobility and continuous measurement because it weighs about 8 kg and has dimensions of 960 mm × 300 mm × 318 mm. It provides full-area coverage of point clouds with a 360° horizontal and 180° vertical (−90° to +90°) field of view. Each sensor operates at a pulse repetition frequency of 30 kHz, and the laser wavelength is 905 nm. The accuracy of spatial positioning is ±3 cm, and the effective range is roughly 100 m when the reflectivity is 20%.

Both the backpack system and the terrestrial laser scanner were utilized in stationary and mobile modes during the data collection process. The terrestrial scanner was positioned about 1.8 m above the ground on a tripod. The tripod's bubble level was used to ensure precise horizontal alignment and prevent geometric distortion or cumulative spatial error in the point cloud data. Five transects with fourteen scanning positions each made up the evenly distributed scanning stations throughout the experimental area (Fig. 1e). Complete coverage with minimal overlap was ensured by the approximate 4 m north-south and 7.5 m east-west distances between neighboring stations. Without color recording, the scanner ran in the "Outdoor High-Density Sampling (10 m)" mode. High-density point clouds that captured minute details of plant architecture were produced by each scan, which took about 8 min. About 9 h were needed to complete the seventy scans. To guarantee accurate registration and dataset comparability, scanning height and angle were kept constant throughout sessions. The LiBackpack D50 system was carried on the operator's back for the mobile scanning process (Fig. 1f), and they followed a pre-planned route that included several closed "triangular" trajectories. For sequential scanning, the entire field was split into two sections. To guarantee steady SLAM operation and high-density data collection, the operator kept their walking speed between 0.6 and 0.8 m/s. Data collection took about 15 min for each section, and both sections were finished in 30 min. Following data collection, SCENE 2019 and LiFuser-BP 1.1 software were used to register, stitch, and filter the raw point clouds, producing high-quality point cloud data in PLY format. Table 1 displays a comparison of sensor specifications.

**Table 1:** Technical parameter comparison for sensors.

Parameter	FARO Focus S	LiBackpack D50
Laser wavelength (nm)	1550	905
Field of view (°)	H: 360° V: 300°	H: 360° V: - 90° ∼ 90°
Detection range at (20% ref)	0.60-100 m	100 m
Mounting height	1.8m	1.2 m
Data acquisition mode	Fixed-point scanning	Mobile triangular-path
Data acquisition duration	8h	0.5h
3D reconstruction algorithm	ICP-based global registration	LiDAR-IMU SLAM
Data resolution	±0.30 mm (10 m @ 90% ref)	30 mm

To fully assess wheat development, 3D point cloud data was collected at four crucial growth stages: joining (March 30), flowering (April 27), grouting (May 6), and maturity (May 20). For every growth stage, both the backpack system and the terrestrial laser scanner were employed.

### 2.3 Preprocessing of the 3D point clouds

Numerous unrelated background points that were unhelpful for wheat trait analysis and would obstruct the precise extraction of canopy structural features were present in the raw 3D point clouds that were acquired from the LiDAR acquisition systems. These background points had to be successfully eliminated to guarantee analytical accuracy. A suitable point cloud segmentation technique can significantly increase processing efficiency, while traditional manual clipping is labor-intensive, subjective, and time-consuming. Ground surfaces were the main source of background noise in this investigation. Although plane-fitting algorithms are frequently used for ground extraction, these techniques presume a level and smooth surface. This assumption is broken in actual field settings by low-growing weeds, footprints, uneven soil surfaces, and soil clods. These non-target features were also recorded due to the high spatial resolution of the terrestrial laser scanner, which made it impossible for traditional plane detection to distinguish between ground and canopy points. Standard segmentation techniques are inappropriate for this study since expanding the detection radius would eliminate these features while also running the risk of eliminating legitimate wheat points.

**Fig. 2:**
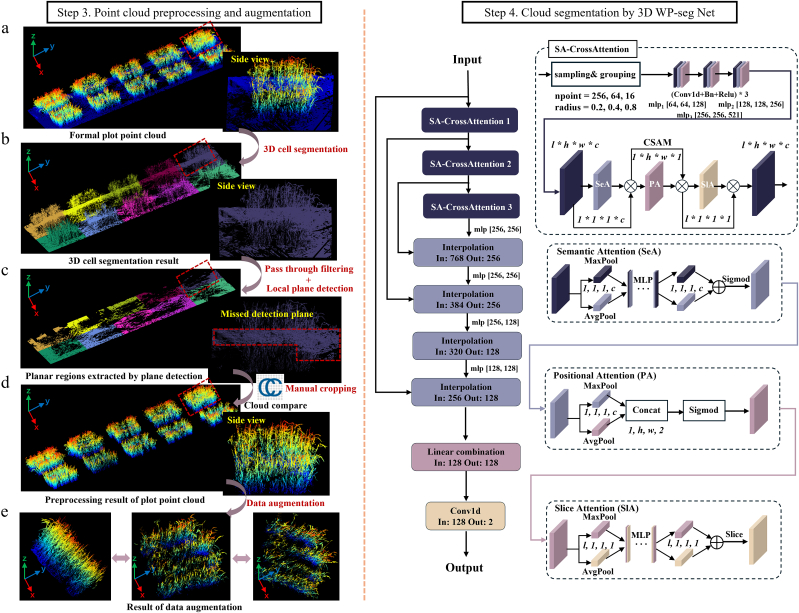
Workflow for preprocessing 3D point clouds. (a) Formal 3D point cloud. (b) Segmentation at plot level. (c) Detection from the ground plane. (d) The canopy point cloud that has been preprocessed. (e) Visualization of data augmentation results applied to the training set.

A specific point cloud preprocessing technique was used to separate background points from wheat canopy points to overcome these constraints. Because of the volume of data and the difficulty of modifying the 3D view, direct manual clipping of the original full-resolution point cloud is difficult and can result in inaccurate annotations. To increase the accuracy and efficiency of annotation, a preprocessing workflow was implemented. Specifically, a block-segmentation algorithm was used to first separate the raw point cloud of the entire planting region (Fig. 2a) into distinct plot-scale subsets. In accordance with the field layout (5 × 2), minimum and maximum coordinates were calculated along the planting direction and its perpendicular direction. The entire dataset was then divided into 10 plot-level point clouds through interpolation (Fig. 2b). Only non-ground points were kept as potential canopy points after ground points were removed for each plot using a radius-based plane detection technique with a 0.2 m search radius (Fig. 2c). The final canopy point cloud for each plot was then obtained by manually refining the remaining background artifacts resulting from plane detection errors using CloudCompare (Fig. 2d). The manual refinement was performed by two independent annotators, with each plot taking approximately 10-15 min. To ensure consistency, all annotations were cross-checked, and a random subset of 10% of plots was annotated independently by both annotators, yielding an average inter-annotator agreement of over 95%. To minimize bias, only points that were clearly background noise were removed, ensuring that low canopy points were preserved. For one N0 and one N240 group, this preprocessing workflow was used for both the jointing and maturity stages, resulting in 480 preprocessed plot-level point clouds.

### 2.4 Building the dataset for point cloud segmentation

To improve the segmentation network's resilience and generalization ability, the 480 preprocessed 3D point clouds were first divided into training, validation, and test sets in a 7:2:1 ratio, yielding 336, 96, and 48 samples, respectively. To minimize potential bias, samples from the same wheat variety were distributed across different subsets. Data augmentation—comprising random rotations in three dimensions, translations along the coordinate axes, scaling with varying factors, Gaussian noise, and probabilistic random point dropout—was applied only to the training set, expanding it to 1680 samples. The validation and test sets remained unchanged to ensure unbiased evaluation. Representative examples of the original and augmented point clouds are shown in Fig. 2e.

### 2.5 3D WP-seg net architecture

To address the issues with wheat plot point clouds, such as structural complexity, background noise, non-uniform spatial distribution, and scale variations, a 3D point cloud segmentation network called 3D WP-seg Net was created using the PointNet++ architecture[36]. The traditional PointNet++ architecture has limitations in modeling complex inter-plant geometries and capturing fine structural boundaries between wheat plants and the ground, despite its ability to process a wide range of point cloud scenarios. To overcome these limitations, we introduce the SA-CrossAttention module, which replaces the conventional Set Abstraction (SA) layers. Unlike a simple replacement, the SA-CrossAttention module enables enhanced contextual feature interaction across different spatial regions by computing cross-attention between local and neighboring point features. This mechanism allows the network to better model long-range dependencies and fine structural details, improving segmentation performance on complex canopy geometries. Furthermore, theoretical analysis suggests that cross-attention facilitates more discriminative feature learning by emphasizing relevant spatial correlations while suppressing background noise, leading to more accurate delineation of wheat plants from the ground.

While neighborhood aggregation is used in the original SA module to encode local geometric features, single-scale neighborhood aggregation is not enough to capture subtle geometric variations for dense wheat point clouds. After every SA operation, the SA-CrossAttention module integrates a Cross Slice Attention Module (CSAM) [37]. The module greatly improves overall segmentation performance by strengthening contextual dependencies, reducing background interference, improving sensitivity to anisotropic structures, and improving local feature representations through the introduction of cross-slice attention.

Three SA-CrossAttention modules and four Feature Propagation (FP) upsampling modules make up the entire 3D WP-seg Net architecture. The binary point cloud segmentation output is obtained by first processing the input point cloud through the three SA-CrossAttention blocks for hierarchical feature abstraction, then passing it through the four FP modules for feature interpolation and upsampling, and finally mapping it through two Conv1d layers.

A sampling-and-grouping block, a PointNet block, and a CSAM block make up each SA-CrossAttention module. With npoint values of 256, 64, and 16 and radius values of 0.2, 0.4, and 0.8 for the three SA-CrossAttention layers, respectively, the sampling-and-grouping operation employs farthest point sampling (FPS) to choose representative points. Neighborhood point coordinates are converted into a local coordinate system in the PointNet layers with respect to each spherical region's centroid. Conv1d, Batch Normalization, and ReLU operations are used to encode local features. Multi-layer perceptrons (MLPs) with the following structures are then used: [64, 64, 128], [128, 128, 256], and [256, 256, 521]. The CSAM module receives the generated feature sets and uses positional, slice-level, and semantic attention mechanisms to refine them.

Prior to performing MLP operations to extract global semantic importance, the CSAM aggregates feature maps using max-pooling and average-pooling to compute a global semantic attention map $M_{sem}$ (Eqn. (1)):

Eqn. 1 $$M_{sem}\left(F\right)=\sigma \left(MLP\left(MaxPool\left(F\right)\right)+MLP\left(AvgPool\left(F\right)\right)\right)$$

After pooling along the slice and channel dimensions, a positional attention map $M_{pos}$ produced (Eqn. (2)), and positional dependencies are encoded using convolution operations:

Eqn. 2 $$M_{pos}\left(M_{sem}\right)=\sigma \left(conv\left[MaxPool\left(M_{sem}\right);AvgPool\left(M_{sem}\right)\right]\right)$$

Lastly, a Gaussian distribution model (Eqn. (3)) is used to calculate slice attention weights $M_{slice}$ (Eqn. (3)):

Eqn. 3 $$M_{slice}=\sigma \left(N\left(\mu ,\Sigma \right)\right),\mu^{\prime }=W_{\mu }V,P^{\prime }=W_{P}V,D^{\prime }=W_{D}V,\Sigma =P^{\prime }{P^{\prime }}^{T}+D^{\prime }$$

where $W_{\mu }\in R^{l\times l}$, $W_{P}\in R^{lr\times l}$,and $W_{D}\in R^{l\times l}$ are the slice-wise mean (μ′), principal components of the covariance (P′), and diagonal components (D′), respectively.

The FP modules carry out hierarchical feature interpolation and upsampling after feature abstraction. After an MLP with channels [256,256], FP4 combines the original input point cloud with the features that were extracted by the third SA-CrossAttention module. Using corresponding MLPs to refine the feature representations, FP3, FP2, and FP1 gradually fuse their respective SA-CrossAttention outputs with the upsampled features from the previous FP module. The 3D WP-seg Net efficiently handles non-uniform point distributions in wheat canopy point clouds, suppresses background noise, and captures intricate geometric structures through a sequence of feature abstraction, attention-enhanced aggregation, and hierarchical upsampling operations. High-quality binary segmentation outputs are eventually produced by the resulting model, which greatly increases segmentation accuracy and fine-detail extraction.

### 2.6 Model training strategy and assessment criteria

An NVIDIA GeForce RTX 4090 GPU with 24 GB of RAM was used to train the 3D WP-seg Net model using the PyTorch 1.13 framework and CUDA 11.6. With a batch size of four, the training was carried out over 100 epochs. A learning rate scheduler and the AdamW optimizer were used, and the initial learning rate was set to 1 × 10^−3^. The model was validated at the end of each training epoch, which comprised roughly 21,000 iterations, to monitor performance and prevent overfitting.

To comprehensively evaluate the segmentation performance of the 3D WP-seg Net model, four key metrics were used: Precision, Mean Intersection over Union (MIoU), Recall, and F1-score. Precision indicates the model's accuracy in positive classification by calculating the percentage of correctly predicted positive instances among all predicted positive samples. By calculating the intersection-over-union ratio for every class and averaging it across all classes, MIoU assesses the overlap between predicted and ground truth labels. The percentage of true positive samples that the model correctly identified out of all actual positives is known as recall. A balanced indicator of accuracy and completeness in segmentation performance, the F1-score is the harmonic mean of precision and recall.

### 2.7 Software development strategy and 3D phenotypic trait extraction

Five phenotypic traits were extracted at the plot scale using the 3D point clouds segmented by the 3D WP-seg Net. These included two novel structural traits, plot expansion distance (PED) and plot lodging angle (PLA), which were obtained from centroid-based and slice-skeletonization algorithms, and three conventional traits, plot height, canopy area, and canopy volume.

**Fig. 3:**
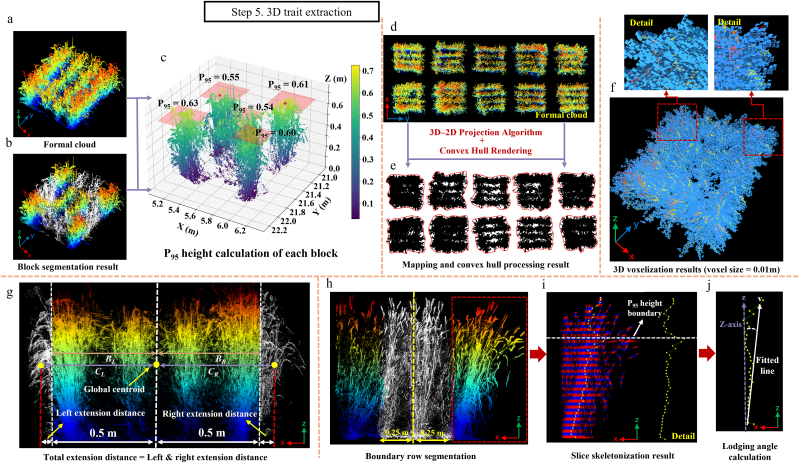
3D phenotypic trait extraction illustration. (a) The initial point cloud at plot level. (b) Plot point cloud sub-block segmentation. (c) Visualization of the P_95_ canopy height proxy. (d) The original point cloud from which the canopy area was extracted. (e) Results of 2D convex hull generation and 3D projection. (f) 3D voxelization of plot point cloud. (g) PED extraction schematic. (h) Boundary-row point cloud extraction. (i) Slice-skeletonization of point clouds with boundary rows. (j) A visual representation of the estimated PLA.

The segmented plot-level point cloud (Fig. 3a) was interpolated along and perpendicular to the planting direction to create nine sub-blocks for plot height to preserve consistency with manual measurement techniques (Fig. 3b). Five sub-blocks were chosen to represent sampling locations in accordance with field measurements: four corners and one center. Each sub-block's P_95_ canopy height proxy was calculated by averaging the top 20% of points with the highest elevation values (Fig. 3c). The estimated plot height was calculated as the mean of these five P_95_ values.

Using a 3D projection mapping algorithm [38], the original point cloud (Fig. 3d) was projected onto the XOY plane for canopy area. Each 2D pixel represented a real 3D area of 0.01 m^2^. The gaps between crop rows occasionally appeared in the resulting 2D projection image. A convex hull algorithm [39] was used to process each connected cluster of projected points in order to take these into consideration (Fig. 3e). To estimate the canopy area, the squared projection coefficient was multiplied by the total number of pixels inside the convex hull.

The plot-level point cloud was subjected to voxelization for canopy volume, using a voxel grid size of 0.01 m (Fig. 3f). The canopy volume estimate for the plot was calculated by multiplying the total number of occupied voxels by the voxel volume.

Traditional methods, which can be sensitive to outliers like widely extended leaves, typically compute the PED as the difference between the maximum and minimum coordinates along the planting direction minus the row spacing. A centroid-based strategy was suggested to increase robustness. Each plot point cloud (Fig. 3g) was divided into three sections (colored and white sections in Fig. 3g) by computing the overall centroid and performing interpolation along the direction perpendicular to the planting rows with a total width of 1 m. The centroids of the two outer sections were computed, and the left and right extension distances between them and the interpolation boundaries were measured along the horizontal direction perpendicular to the planting rows. The total distance of the PED was the sum of these distances (Eqn. (4)).

Eqn. 4 $$PED=\left|\left(C_{L}^{xy}-B_{L}^{xy}\right)\cdot n_{L}\right|+\left|\left(C_{R}^{xy}-B_{R}^{xy}\right)\cdot n_{R}\right|$$

where $C_{L}^{xy}$ and $B_{L}^{xy}$ are the horizontal coordinates of the centroids of the left and right outer sections, respectively; and $C_{R}^{xy}$ and $B_{R}^{xy}$ are the corresponding horizontal coordinates of the interpolation boundaries; and $n_{L}$ and $n_{R}$ are the outward unit vectors perpendicular to the planting rows, indicating the measurement directions of the left and right extensions, respectively.

A slice-skeletonization technique was used for the PLA. After determining the plot point cloud's overall centroid, two boundary rows were extracted using 0.25 m interpolation on each side (Fig. 3h). The centroid of each slice was designated as a skeleton point, and each boundary row was cut into 0.1 m intervals along the depth direction (Fig. 3i). The lodging angle for that boundary row was determined by fitting a straight line to the skeleton points below the P_95_ height threshold and measuring the angle between the line and the vertical axis. The final PLA was explicitly calculated as the average of the lodging angles of the two boundary rows (Eqn. (5)).

Eqn. 5 $$PLA=\frac{1}{2}\left[\arccos\left(\frac{v_{L}\;\cdot z}{\left\|v_{L}\right\|\left\|z\right\|}\right)+\arccos\left(\frac{v_{R}\;\cdot z}{\left\|v_{R}\right\|\left\|z\right\|}\right)\right]$$

where $v_{L}$ and $v_{R}$ are the direction vectors of the fitted lines for the left and right boundary rows, respectively, and z = (0,0,1) is the vertical axis unit vector.

A 3D phenotypic analysis program called 3D Trait Analysis was created utilizing the PyQt5 framework in order to increase the effectiveness of phenotypic parameter extraction and enhance visualization [40]. Multiple scientific computation and visualization libraries, such as Open3D [41]、OpenCV [41]、PyTorch [42] and MMDet3D [43], were integrated into the Python-based software. Stable performance across various operating systems is guaranteed by the system's robust cross-platform compatibility and modular architecture. The program can be launched directly through an executable file without additional compilation or configuration, enabling users to visualize 3D point clouds, conduct interactive analyses, and automatically extract phenotypic parameters efficiently.

### 2.8 Genome-wide association analysis based on phenotypic traits

The Axiom Wheat660 Genotyping Array, a high-density SNP genotyping chip co-developed by Prof. Jizeng Jia's research group at the Institute of Crop Sciences, Chinese Academy of Agricultural Sciences, and Affymetrix Inc., was used in this study to genotype 120 wheat accessions [44] This array detects 630,517 SNP loci, including insertion-deletion variations (InDels) and single-nucleotide polymorphisms (SNPs), and shows more than 95% effective marker coverage with uniform distribution throughout the genome. To ensure the quality of the data, genotype calling and quality control were conducted using Axiom Analysis Suite v5.2 (Affymetrix, Santa Clara, USA). SNPs that had a minor allele frequency (MAF) of less than 0.05 or a missing rate of more than 10% were eliminated. A total of 409,976 high-quality SNP loci were kept for further association analyses following quality control. The IWGSC RefSeq v1.0 wheat reference genome served as the basis for the alignment and annotation of each SNP's physical location. Statistical analyses of phenotypic differences were performed using Statistical Product and Service Solutions (SPSS, version 26.0.0.0; IBM Corp., Armonk, NY, USA). Differences in PED and PLA among different growth stages were assessed using one-way analysis of variance (ANOVA) followed by Tukey's honestly significant difference (HSD) test. Different lowercase letters were used to indicate significant differences among groups at p < 0.05.

GWAS were carried out for two recently defined phenotypic traits: PED and PLA based on the high-quality genotypic dataset in order to find genetic loci linked to phenotypic variation. The rMVP software package with the Fixed and Random Model Circulating Probability Unification (FarmCPU) statistical model, which combines fixed and random effects to increase statistical power and lower false positives, was used to conduct the GWAS analysis. Manhattan plots were used to visualize significant marker–trait associations and highlight loci that exceeded the threshold for genome-wide significance. The Dr.Tom multi-omics data mining platform (https://biosys.bgi.com), created by BGI Genomics, was used to perform functional annotation and biological relevance analysis after candidate genes were found for each significantly associated SNP using the WheatOmics multi-omics database. The molecular mechanisms behind the observed phenotypic variation in wheat structural traits were clarified by these analyses. To identify significant marker-trait associations, a rigorous genome-wide significance threshold was established. Considering the large genome size and extensive linkage disequilibrium inherent to natural wheat populations, applying a strict Bonferroni correction is often overly conservative and may mask valuable loci associated with complex quantitative traits [45]. Therefore, an empirical threshold of -log10(P)≥4.0 was applied to identify suggestive marker-trait associations.

## 3 Results

### 3.1 Utilizing the 3D trait analysis application to gather 3D phenotypic information of the wheat population

**Fig. 4:**
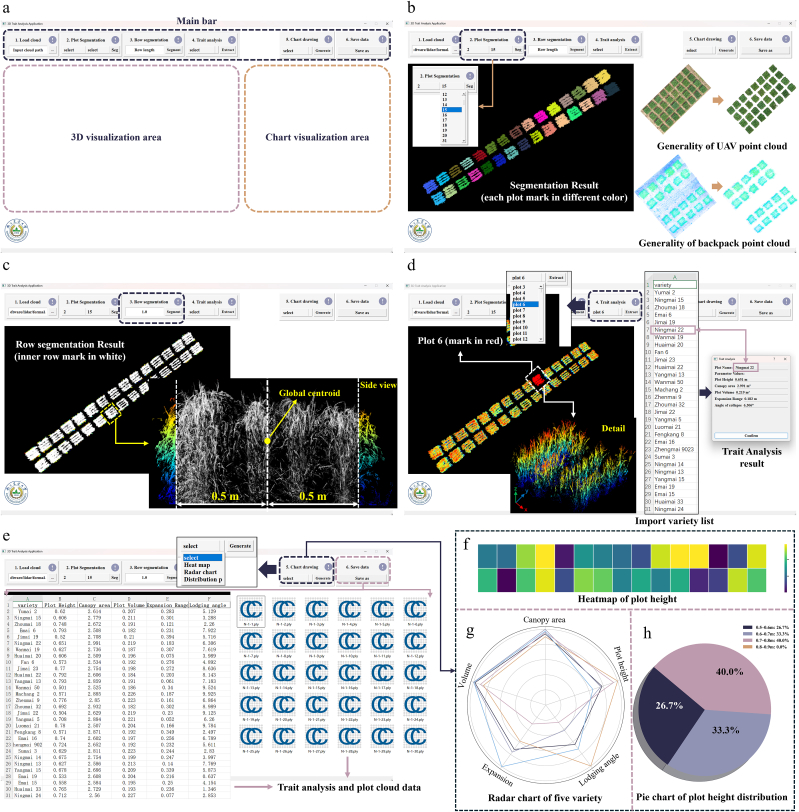
Overview of the 3D Trait Analysis application's functionality. (a) The main functional areas and interface layout. (b) Plot segmentation module illustration. (c) The outer-row segmentation function is demonstrated. (d) Plot-level phenotypic trait extraction function visualization. (e) Demonstration of the data export and chart visualization module. (f) Heatmap of phenotype. (g) Radar chart for phenotypes. (h) Pie chart of phenotypic distribution.

A main toolbar, a point cloud visualization interface, and a chart display area comprise the three main functional sections of the 3D Trait Analysis application (Fig. 4a), which was designed with a modular architecture. The main toolbar comprises six functional modules: 3D point cloud data loading, plot segmentation, outer-row extraction, trait analysis, chart visualization, and data export. It integrates the entire workflow from data import to result export. Users can import one or more point cloud datasets (such as those in.pcd or.ply formats) into the 3D point cloud loading module. Once loaded, the visualization interface allows for flexible inspection through mouse-based zooming and panning, as well as interactive 3D viewing from various perspectives. Users specify the number of planting rows and columns in the plot segmentation module (Fig. 4b), and the program automatically divides the raw point cloud in accordance with those settings. The preloaded 3D WP-seg Net model then processes the segmented subsets, performing segmentation and visualizing the resulting plot-level point clouds. This module works with 3D data from backpack LiDAR scanning systems as well as UAV-based multi-view imaging. Targeted analysis of outer planting rows is made possible by the outer-row extraction module (Fig. 4c), which segments the extended border regions according to the user-specified plot width. Plot- and variety-level phenotypic analyses are possible with the trait extraction module (Fig. 4d). The system creates a mapping between plot coordinates and corresponding variety names based on the row and column configuration that users specify after they first import a variety index table. A specific trait analysis window displaying the variety name and its five extracted phenotypic parameters appears when a target plot number is chosen from the dropdown list. To improve identification, the chosen plot is simultaneously highlighted in red in the 3D visualization interface. Several statistical graphics, such as pie charts (Fig. 4h), radar charts (Fig. 4g), and phenotypic heatmaps (Fig. 4f), can be generated using the chart visualization module. Each of these visualizations shows the distribution ranges of phenotypic parameters, inter-varietal trait differences, and growth variations among varieties within the same developmental stage. In order to facilitate record management and subsequent data analysis, the data export module (Fig. 4e) saves the processed 3D plot data and the associated phenotypic results in numbered files to a specified output directory.

### 3.2 Evaluation of 3D WP-seg Net's performance compared to other segmentation models

Six popular point cloud segmentation architectures: PointNet++, PointTransformer v3 [46], PointTransformer v1 [47], OneFormer3D [48], DGCNN [49] and PointConv [50] were chosen as benchmark models for comparative experiments in order to confirm the efficacy of the suggested 3D WP-seg Net model. To guarantee experimental fairness, all models were trained using the same setups. Four primary quantitative metrics: Precision, MIoU, Recall, and F1-score were used for performance evaluation to thoroughly analyze segmentation performance (Table 2). The results showed that in the wheat 3D plot segmentation task, the 3D WP-seg Net model outperformed the comparison models and showed its superior segmentation capability, achieving the highest scores across all four indicators.

**Table 2:** Comparison of assessment indicators in different models.

Model name	Precision	MIoU	Recall	F1-score
3D WP-seg Net (Ours)	95.1%	92.9%	93.6%	94.3%
PointNet++	93.8%	90.4%	92.3%	93.1%
PointTransformer v3	93.7%	91.3%	92.2%	93.3%
PointTransformer v1	92.6%	90.8%	91.8%	92.1%
OneFormer3D	92.3%	91.0%	92.0%	91.7%
DGCNN	91.8%	90.2%	91.3%	91.6%
PointConv	91.9%	90.1%	91.2%	91.4%

**Fig. 5:**
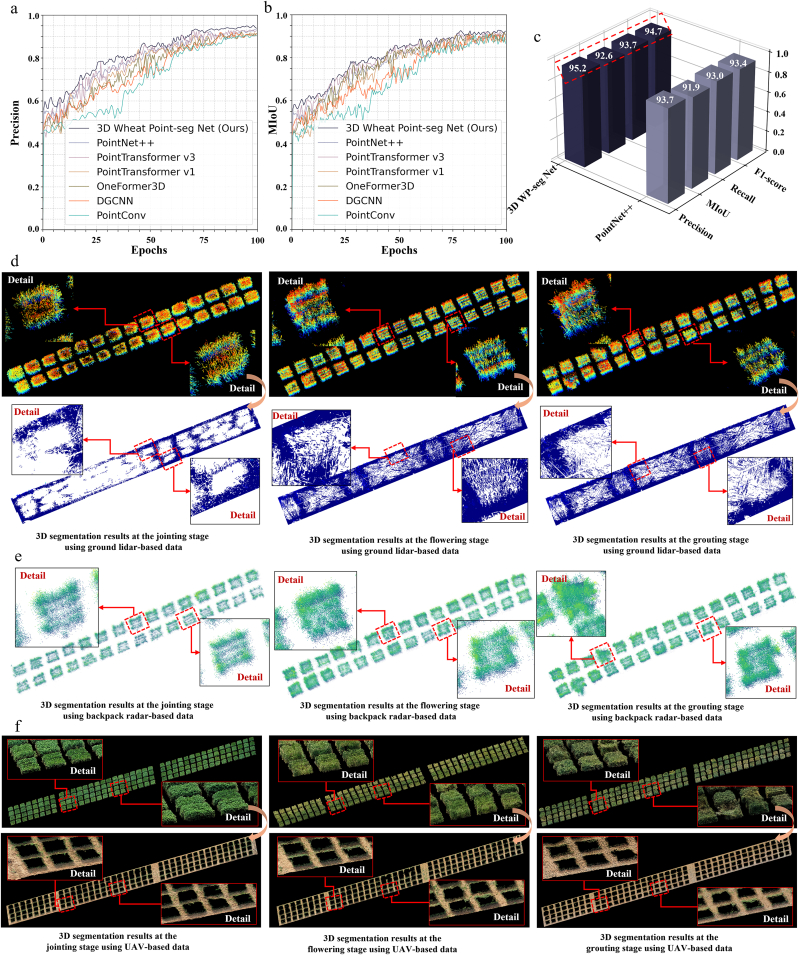
Evaluation of model performance. (a) The precision curve over training periods. (b) MIoU curve over training periods. (c) Ablation study findings. (d) Segmentation results using data from terrestrial laser scanners at critical growth stages. (e) Segmentation results using data from BLS at critical growth stages. (f) Segmentation results using UAV-derived 3D reconstruction data at critical growth stages.

Each model was trained for 100 epochs during the segmentation experiments, and the trends of the Precision and MIoU values over the training epochs were plotted (Fig. 5a and b). The findings demonstrated that after about 70 epochs, the 3D WP-seg Net achieved a stable performance plateau, following which its Precision and MIoU values remained constant. DGCNN and PointConv continued to show performance fluctuations even after 100 epochs, whereas the PointNet++, PointTransformer v3, and PointTransformer v1 models required roughly 80 and 85 epochs, respectively, to reach comparable stability. The OneFormer3D model, on the other hand, needed approximately 85 and 90 epochs to stabilize its Precision and MIoU values. These results further confirm the robustness and efficacy of 3D WP-seg Net in 3D wheat plot segmentation tasks by showing that it not only achieves higher segmentation accuracy but also exhibits improved training efficiency and convergence stability compared to existing architectures.

### 3.3 Ablation tests demonstrating the value of the modified modules

Four performance indicators: Precision, MIoU, Recall, and F1-score were used in an ablation study to assess the contribution of the recently added modules. The results (Fig. 5c) showed that the 3D WP-seg Net's performance was significantly enhanced across all evaluation metrics by substituting the SA-CrossAttention layer for the original set abstraction layer. These results verify that the network's segmentation capability is improved by the new and altered components. The ablation results provide strong evidence that the architectural improvements introduced in this study have practical applications to improve segmentation accuracy in complex wheat plot point cloud data.

### 3.4 3D WP-seg Net's high generalization capability across various sensor-derived 3D data

The 3D WP-seg Net model's generalization ability was evaluated using point cloud datasets from three distinct sensing modalities: UAV-based multi-view 3D reconstruction, backpack laser scanning (MLS), and terrestrial laser scanning (TLS). The 3D WP-seg Net was used to segment representative plot-level point clouds from the jointing, flowering, and grouting stages (Fig. 5d–f). Precision, MIoU, Recall, and F1-score were used to evaluate segmentation performance (Table 3). The segmentation accuracy of the TLS-derived point clouds was the highest among the three data sources, followed by UAV-based reconstructions, and the lowest among the MLS-derived point clouds (see Table 4).

**Table 3:** Comparison of assessment indicators in different sensors.

Data source	Precision	MIoU	Recall	F1-score
Ground-based lidar	93.1%	91.7%	92.2%	92.4%
Backpack radar	88.3%	86.4%	87.6%	88.1%
UAS	92.5%	90.2%	90.8%	91.1%

**Table 4:** Description of potential regulatory genes.

Trait	Gene ID	-log 10 (P)	Description
PLA	TraesCS2D02G541700	4.23	F-box family protein
PLA	TraesCS2D02G554600	4.81	F-box family protein
PLA	TraesCS3A02G465500	4.28	F-box family protein
PLA	TraesCS3A02G446100	4.36	F-box family protein
PLA	TraesCS7A02G381900	4.07	F-box family protein
PED	TraesCS1A02G379700	4.12	Myb transcription factor
PED	TraesCS2A02G498900	5.26	Translation initiation factor IF-2
PED	TraesCS4A02G028900	4.31	BHLH family transcription factor
PED	TraesCS4A02G345000	5.04	Expansion

These variations are a direct result of the point cloud sources' differing levels of precision and noise. The segmentation network can extract more distinct structural features and attain higher accuracy and recall thanks to TLS's highest measurement fidelity and most consistent point quality. High-quality 3D data is also produced by UAV-based multi-view reconstruction; however, because camera-based depth estimation has inherent limitations, its performance is marginally worse. Comparatively speaking to the other two approaches, segmentation performance is adversely affected by MLS data's greater susceptibility to measurement noise, accumulated pose drift, and scanning inconsistencies.

All things considered, this experiment demonstrates the 3D WP-seg Net's resilience and versatility across a range of sensing technologies and data types. The findings also show that the model retains dependable segmentation performance even when working with heterogeneous 3D point cloud sources, and they theoretically support the choice of suitable sensors in real-world applications.

### 3.5 Assessment and analysis of trait accuracy

**Fig. 6:**
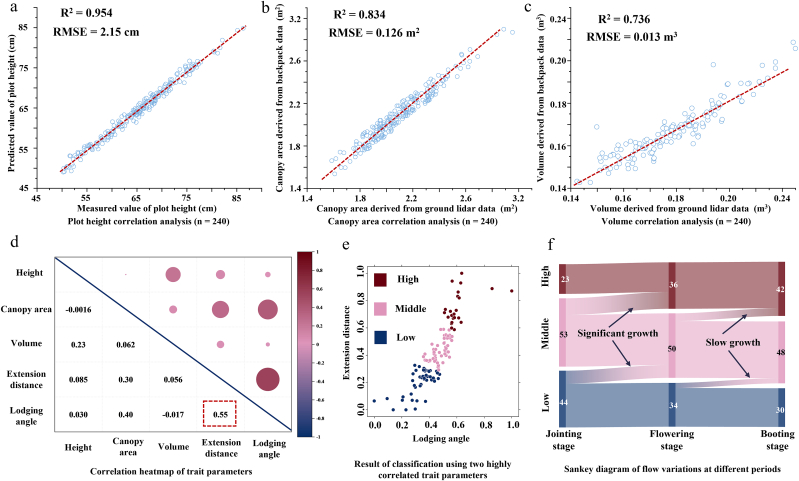
Assessment of trait accuracy and prediction of classification. (a) Correlation analysis of plant height. (b) Canopy area correlation analysis using data from TLS and MLS. (c) Plot volume correlation analysis using data from TLS and MLS. (d) Five phenotypic traits' correlation analysis heatmap. (e) Classification results according to two traits that are highly correlated. (f) A Sankey diagram that depicts the flow of classification across various growth stages.

In order to perform a correlation analysis of various phenotypic traits, 240 plot samples were chosen at random from 120 wheat varieties that were planted at the Baimai Teaching and Research Base between 2022 and 2023. The suggested method's accuracy and stability in estimating different structural parameters were confirmed by contrasting the model's predicted values with manually measured values. Plot height (Fig. 6a) showed a high degree of consistency between the measured and predicted values, with a root mean square error (RMSE) of only 2.15 cm and a coefficient of determination (R^2^) of 0.954. This outcome shows that the technique can accurately and consistently record minute variations in wheat plant height.

Since there were no manual measurements available, the study used both TLS and MLS data from the same plots for modeling and fitting with the aim to determine canopy area and plot volume. The findings indicated that the model accurately captures the spatial distribution of the canopy structure, with an R^2^ for canopy area of 0.834 and an RMSE of 0.126 m^2^ (Fig. 6b). With an R^2^ of 0.736 and an RMSE of 0.013 m^3^, the correlation for plot volume estimation (Fig. 6c) was significantly lower than that for canopy area. Notably, the volume determined using the TLS data was typically greater than the MLS system's estimates. Variations in sensor resolution and imaging precision are the primary causes of this discrepancy.

The platform's height variability, limited scanning angles, and relatively low point cloud density limit the MLS system's ability to capture detailed features of the plant top and canopy interior, despite its mobility and high operational efficiency. These restrictions may cause some branch and leaf data to be overlooked, which would understate the total volume of the plant. Furthermore, under complicated field terrain and wind conditions, MLS data are susceptible to point cloud distortions or overlapping errors, which can result in minor deviations during model fitting. However, the TLS system's steady scanning angle and higher point cloud density enable more accurate measurements of the plant canopy and internal structures, which in turn produce more accurate volume estimates.

### 3.6 Predicting classification using highly correlated trait parameters

Five phenotypic traits were analyzed for correlation in this section at a significance level of P < 0.01; combinations of highly correlated traits were chosen for classification prediction. PED and PLA have a significant positive correlation, according to the correlation analysis results (Fig. 6d). The study normalized the PED and PLA parameters after choosing 120 plot samples from the N240 treatment at the jointing stage based on this correlation. Three-class classification (Fig. 6e) was carried out using a random forest algorithm [51], grouping the plots into high, medium, and low categories. (The "high, medium, low" categories are relative rankings rather than absolute levels because the study is focused on high-yielding varieties.) Data from the flowering and grouting stages were classified using the same process, and category transitions between various growth stages were visualized using Sankey diagrams (Fig. 6f). The number of plots moving from low to medium and from medium to high categories from the jointing to flowering stages increased significantly, according to the results, and the trend from flowering to grouting continued to rise.

The physiological alterations of wheat plants at various growth stages are consistent with this pattern. Agronomically speaking, the time between jointing and flowering is crucial for the change from vegetative to reproductive growth because it is when the plant stem rapidly elongates, the photosynthetic area of the leaves increases, and the leaf angle becomes more ideal for light interception. The loosening of the canopy structure caused by leaf expansion results in an increase in PED and a slight increase in the PLA. Grain development in the upper plant and the growing weight of the spikes further tax the stem after it enters the grouting stage, which causes the PLA to slightly increase. Thus, both PED and PLA typically exhibit an upward trend from jointing to flowering and then to grouting. This finding supports previous studies on the dynamic structural changes in wheat populations and confirms that the classification prediction method used in this study provides dependable technological support for dynamic crop phenotyping monitoring by accurately capturing the temporal changes in plant morphological parameters.

### 3.7 Predicting candidate genes using phenotypic parameters

**Fig. 7:**
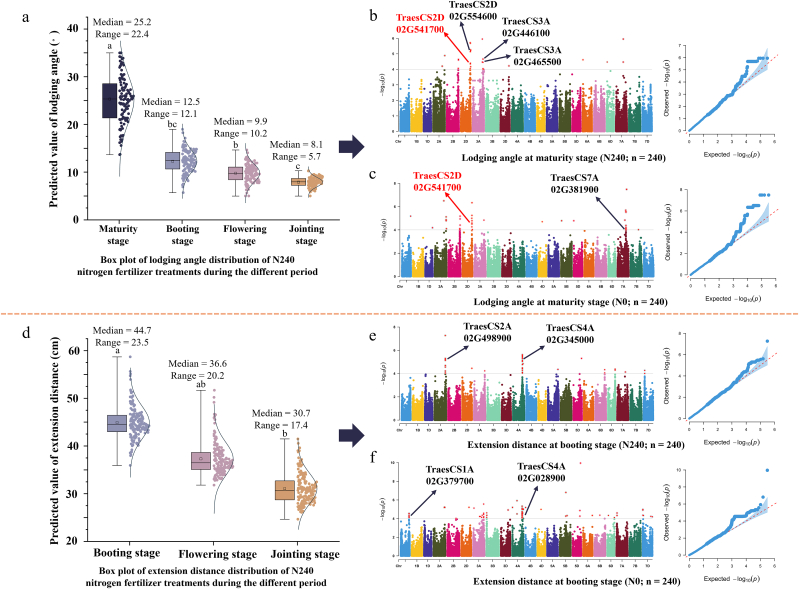
GWAS results and phenotypic variation of PLA and PED. (a, d) Boxplots of PLA and PED under the N240 treatment at different growth stages, respectively. Different lowercase letters indicate significant differences among groups based on one-way ANOVA followed by Tukey's HSD test at p < 0.05.(b-c, e-f) Manhattan plots for PLA at maturity and PED at the grain-filling stage under the N240 and N0 treatments, respectively. The black horizontal line indicates the empirical significance threshold of -log10(P)≥4.0, which was used to identify suggestive marker–trait associations. The red line represents the reference line with R^2^ = 1.

The effectiveness of the suggested trait extraction algorithm was validated in this section by conducting GWAS using the two phenotypic parameters: PLA and PED to find possible candidate genes. PLA boxplots for 240 wheat varieties under N240 and N0 nitrogen treatments are shown in Fig. 7a. The plots span four representative growth stages: jointing, flowering, grouting, and maturity. All four stages' parameters showed normal distributions, but the maturity stage showed a noticeably higher lodging angle than the previous stages. Fig. 7b–c displays the GWAS results. Numerous significantly associated SNPs were found on chromosomes 2D, 3A, and 7A in the Manhattan plots, with multiple significant loci forming continuous regional clusters. There were clear "peak" and "band-like" patterns in the signals that were above the significance threshold. The QQ plots, on the other hand, showed that the observed SNP distributions were mostly in agreement with the predicted theoretical curves, with only minor variations in the tail regions. This suggests that the detected association signals were extremely reliable and that the GWAS results were unaffected by systematic bias or false-positive inflation brought on by population structure. PLA may be regulated by five genes found on chromosomes 2D, 3A, and 7A, according to gene annotation results. The same putative gene loci were found on chromosome 2D in both N240 treatment groups. The annotation indicates that each of the five potential genes is a member of the F-box family. F-box proteins, which are essential parts of the SCF (SKP1–Cullin–F-box) complex in the ubiquitination pathway, usually mediate the selective degradation of target proteins to control stress responses, hormone signaling, and plant growth and development [52]. According to earlier research, members of the F-box family are essential for the physiologic processes of cell wall biosynthesis, plant architecture formation, and mechanical strength regulation—all of which are intimately linked to lodging resistance [53]. As a result, the strong correlation found in this study between these F-box genes and PLA raises the possibility that they contribute to the development and control of this trait by affecting internode elongation, stem wall mechanical characteristics, or hormone regulatory networks (such as auxin/gibberellin signaling), offering important new information on the genetic foundation of traits related to lodging.

The jointing, flowering, and grouting stages under both N240 and N0 nitrogen treatments were chosen for analysis because many varieties displayed large lodging angles at maturity, which reduced the segmentation accuracy of horizontally distributed point clouds. Fig. 7d displays boxplots of PED for each of these stages. Four potential genes on chromosomes 1A, 2A, and 4A were identified by GWAS results (Fig. 7e and f). The MYB transcription factor, which is encoded by the gene found on chromosome 1A, is essential for controlling hormone signaling pathways, cell wall biosynthesis, and cell elongation. It may also have an impact on the horizontal expansion of plots across various growth stages. By regulating cell proliferation and tissue growth rates, the translation initiation factor IF-2, a protein intimately linked to translational efficiency, is encoded by the gene on chromosome 2A and may indirectly affect canopy structural expansion [54]. Two genes were found on chromosome 4A that encode a basic helix–loop–helix (bHLH) transcription factor and an expansion protein, respectively. Expansion directly promotes radial and horizontal tissue growth by acting as a major regulator of cell wall elongation and loosening [55], while members of the bHLH family play significant roles in the formation of canopy architecture and are extensively involved in the regulation of hormone response, leaf expansion, and internode elongation [56]. Given their strong correlations with PED variation from the jointing to grouting stages and their close associations with cell elongation, cell wall remodeling, and growth regulatory networks, these four genes may contribute to the genetic regulation of this trait by affecting canopy configuration and tissue expansion dynamics.

## 4 Discussion

### 4.1 Benefits of the 3D WP-seg net model for 3D segmentation at the wheat plot level

Compared to current point cloud segmentation techniques, the suggested 3D WP-seg Net point cloud segmentation network exhibits significant advantages. Point cloud libraries are the primary component of conventional 3D point cloud processing methods [57]. For example [58], created an enhanced density-based clustering technique based on point cloud density features, while [59] used the RANSAC plane-fitting algorithm to extract ground points. However, their approach primarily concentrated on extracting upper canopy points and showed poor recognition ability for lower structures due to the limited imaging angle and coverage range of RGB-D devices, making it challenging to achieve accurate segmentation at the population plot level. Deep learning-based 3D point cloud segmentation networks, on the other hand, do not require complicated parameter tuning, allowing raw point clouds to be processed from beginning to end and yielding accurate segmentation results. These techniques are quickly replacing more conventional point cloud processing methods due to their automatic feature extraction and increased adaptability [60]. used PointNet++ for plot-level classification, while [61] used a CNN-based framework to segment planar and non-planar point clouds of wheat plots. However, the use of multi-view reconstructed point clouds produced by UAVs in both studies limited their capacity to conduct fine-grained analysis of wheat plot phenotypes. Since there aren't any segmentation models specifically made for plot-level point clouds obtained using terrestrial LiDAR, this study created the 3D WP-seg Net, a segmentation network made for this kind of situation. To improve the model's ability to capture and depict subtle 3D structural information, the original SA layer was swapped out for an SA-CrossAttention module.

### 4.2 Genetic Determinants of 3D population-level traits and their biological significance

The integration of high-resolution 3D phenotyping with genome-wide association studies (GWAS) enables precise dissection of the genetic architecture underlying complex plant structural traits. Significant SNP clusters associated with PLA were identified on chromosomes 2D, 3A, and 7A, providing strong validation of the efficacy of our 3D WP-seg Net framework. Notably, loci on chromosomes 2D and 3A co-localize with previously reported QTLs controlling stem thickness and structural integrity in wheat [62]. Among the candidate genes, F-box family proteins likely serve as key regulators; as components of the SCF ubiquitin ligase complex, they mediate the targeted degradation of repressors within the auxin and gibberellin signaling pathways, thereby modulating cell wall remodeling and mechanical strength [52].

Moreover, candidate genes influencing ED were identified on chromosome 4A, including an expansion protein and a bHLH transcription factor, offering novel insights into canopy dynamics. Expansion proteins are known to facilitate cell wall loosening, directly promoting tissue expansion [55]. While individual architectural traits, such as tiller angle, are fundamental, our results suggest that population-level “extension distance” is governed by distinct genetic loci. This indicates that the 3D traits extracted by our pipeline capture higher-order architectural phenotypes, encompassing not only individual growth angles but also the coordinated spatial distribution and canopy interactions within dense wheat populations.

### 4.3 Benefits and drawbacks of the slicing algorithm for plot-level 3D data processing

**Fig. 8:**
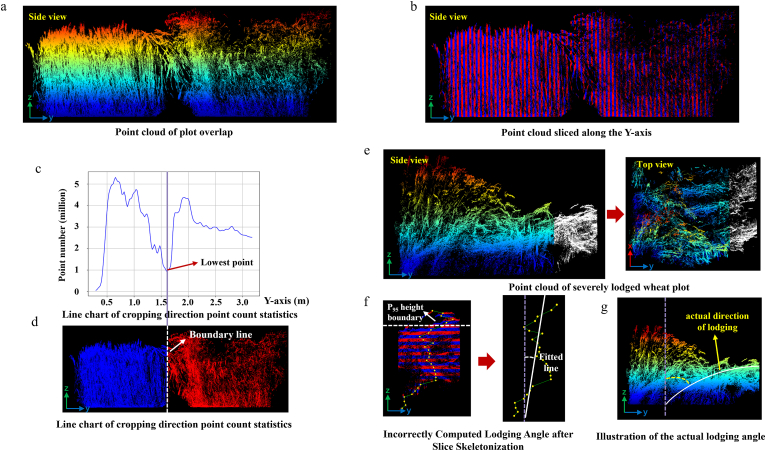
Benefits and drawbacks of the slicing algorithm in various contexts. (a) Original point cloud of overlapping plots. (b) Results of horizontal slicing for overlapping plots. (c) Distribution curve of point cloud numbers for each horizontal slicing layer. (d) Results of segmenting overlapping plots according to point cloud numbers. (e) Original point cloud of plots with severe lodgment. (f) Illustration of the lodging angle that was computed incorrectly using the slicing-skeletonization algorithm. (g) Example of the real lodging angle.

There are advantages and disadvantages to the slicing algorithm that was employed in this study to analyze the PLA. Point clouds from nearby plots may overlap when lodging gets severe during the wheat maturity stage (Fig. 8a). Plots that overlap in these situations may be mistakenly interpreted as a single plot in later analysis. Slices of 0.1 m thickness were used along the planting direction as part of a slicing-based strategy to solve this problem (Fig. 8a). A Y-axis-point count curve was created by counting the points in each slice layer (Fig. 8c). The minimum point density in the Y-axis range of 1.5–2.0 m was used as the dividing line to separate overlapping plots because each plot is 1.5 m long and there is a 0.5 m gap between plots (Fig. 8d). When it came to mild to moderate plot overlap, this approach worked well. However, the lodging angle estimated by the slicing-skeletonization algorithm (Fig. 8f) differed significantly from the actual lodging angle (Fig. 8g) in cases of severe lodging (complete collapse; Fig. 8e).

Under severe lodging, inner-row plants bend considerably toward outer rows, causing a large amount of spatial overlap and this deviation. This causes the height, orientation, and spatial distribution of point clouds from various rows to become entangled. Under these circumstances, the point cloud's general geometric skeleton no longer correctly depicts the actual growth direction of individual plants or rows. Because of this bias, the main axis vector that is taken from the mixed point cloud introduces systematic errors and yields an estimated lodging angle that is significantly different from reality.

In conclusion, the slicing algorithm works well for mild to moderate plot overlaps, especially when plant structures are still mostly intact and lodging is less severe during the jointing, flowering, and grouting stages. Plot boundaries can be accurately identified and the subsequent lodging angle extraction can be guaranteed thanks to its density-based segmentation strategy along the longitudinal axis. But by its very nature, this approach depends on plants preserving comparatively autonomous row structures in three dimensions. Extensive overlap and spatial mixing hinder the precise recovery of the actual plant geometry at the maturity stage, when lodging becomes extreme. As a result, the algorithm exhibits definite limitations in these circumstances. All things considered, the slicing algorithm performs admirably in most real-world scenarios; however, to increase the accuracy of lodging angle estimation in severe lodging situations, it must be integrated with more reliable point cloud segmentation or structural recognition methods.

### 4.4 Future perspectives

Although there is still opportunity for improvement, the suggested 3D point cloud segmentation network has shown good generalization ability and robustness in wheat population-scale phenotypic analysis. To assess the network architecture's stability and adaptability under various plant architectures, canopy configurations, and density conditions, it could first be applied to other crop populations, such as maize, rice, and soybean. Future research will focus on optimizing the network architecture based on statistical differences in cross-crop point cloud features because different crops show significant variations in plant height, panicle structure, and morphology. To enable high-precision 3D phenotypic extraction across various agricultural scenarios, improvements could include unified cross-platform training schemes, multi-scale feature fusion strategies, and lightweight attention modules.

Finer organ-scale characteristics (such as 3D spike morphology, spike length, curvature, and grain spatial distribution) are still difficult for terrestrial LiDAR systems to detect, despite this study's strong performance at the population trait level. High-resolution organ-level analyses are hampered by imaging limitations such as limited scanning distance, angular occlusion, and uneven reflectance brought on by the roughness of the panicle surface, which produce sparse, noisy, and incomplete point clouds. A gantry-based 3D scanning system with close-range, high-density sampling, where active sensor trajectory control reduces occlusion and increases spike-level point cloud density, may be developed in the future. Furthermore, combining multi-view image reconstruction or high-resolution structured light techniques may result in more comprehensive and detailed 3D spike models appropriate for organ-level phenotypic quantification, opening up a new technological avenue for high-throughput analysis of wheat spike traits.

## 5 Conclusion

This study proposed a 3D trait extraction method for wheat populations with multiple growth stages using terrestrial LiDAR data. First, 3D WP-seg Net, a 3D segmentation network created especially for wheat plot scenarios, was proposed. It performed well, scoring 95.1% in Precision, 92.9% in MIoU, 93.6% in Recall, and 94.3% in F1-score. Then, two novel phenotypic traits: ED and lodging angle were introduced by combining slicing-skeletonization and centroid localization techniques. To further support the processing of multi-sensor point clouds and trait analysis, the 3D Trait Analysis software platform was developed. Ultimately, nine candidate genes that were significantly linked to these two novel traits were found using genome-wide association analysis.

The application accuracy of terrestrial LiDAR data in wheat population analysis was successfully increased by the 3D trait extraction method suggested in this study. Furthermore, this study identified candidate genes linked to important wheat growth traits by introducing novel phenotypic indices and genome-wide association analysis, offering fresh research instruments and theoretical underpinnings for precision agriculture and wheat breeding.

## Credit authorship author contribution statement

Honghao Zhou: Writing – editing, Writing – original draft, Methodology, Investigation, Conceptualization, Data curation, Formal analysis, Software, Validation, Visualization.

Bingxi Qin, Chunmei Guo, Jingrong Zang, An Wang, Yin Li, Zijun Pei, Qi Sun, and Qiang Zheng: Writing – review & editing, Supervision.

Jiawei Chen, Qing Li, and Dong Jiang: Writing – review & editing, Writing – original draft, Methodology, Investigation, Conceptualization, Data curation, Formal analysis, Resources, Software, Validation, Visualization.

## Funding

This research was supported by the projects of the National Key Research and Development Program of China (2023YFD2300201), Jiangsu Provincial Natural Science Foundation (Youth Program) of China (BK20251511), National Natural Science Foundation of China (32021004), Jiangsu Innovation Support Program for International Science and Technology Cooperation Project (BZ2023049), Lhasa City Science and Technology Program Projects (LSKJ202520), the Key R&D Project of Shandong Province (2024TZXD070), Shandong Province First-class Discipline Construction '811′ Project, the earmarked fund for CARS-03, and the collaborative Innovation Center for Modern Crop Production co-sponsored by Province and Ministry (CIC-MCP).

## Declaration of competing interest

The authors declare that they have no known competing financial interests or personal relationships that could have appeared to influence the work reported in this paper.

## Data Availability

The source code is distributed under the Creative Commons Attribution 4.0 international license, permitting academic use, distribution, reproduction in any medium, provided you give appropriate credit to the original authors and the source, provide a link to the Creative Commons license, and indicate if changes were made. Unless otherwise stated, the Creative Commons Public Domain Dedication (http://creativecommons.org/licenses/by/4.0) waiver applies to the data and results made available in this paper. The source code, testing data, and other datasets supporting the results presented here are available at https://github.com/AI-PhenoLab/3D-WP-seg-Net.
